# Vitamin A is associated with all-cause mortality in patients with chronic kidney disease: a population-based cohort study

**DOI:** 10.3389/fnut.2024.1469844

**Published:** 2024-12-04

**Authors:** Yunxia Feng, Yuan Li, Shuo Chen, Na Hu, Dan Liao

**Affiliations:** ^1^Department of Nephrology, Mianyang Central Hospital, University of Electronic Science and Technology of China, Mianyang, China; ^2^Department of Anesthesiology, Affiliated Hospital of Zunyi Medical University, College of Anesthesiology, Zunyi Medical University, Zunyi, China; ^3^Department of Anesthesiology, Mianyang Hospital of Traditional Chinese Medicine, Mianyang, China; ^4^Department of Critical Care Medicine, Mianyang Central Hospital, University of Electronic Science and Technology of China, Mianyang, China

**Keywords:** chronic kidney disease, vitamin A, all-cause mortality, NHANSE, cohort study

## Abstract

**Introduction:**

The association between serum vitamin A (VA) levels and outcomes in chronic kidney disease (CKD) patients remains unclear.

**Methods:**

This was a population-based cohort study. CKD participants from the National Health and Nutrition Examination Survey (NHANES) database were included for analysis. The primary outcome was all-cause mortality. Person correlation analysis and Cox regression models were used to assess the relation between serum VA levels and all-cause mortality among individuals with CKD.

**Results:**

There were 689 participants included in this study. The serum VA level was 2.45 ± 1.06 μmol/L. The overall mortality was 43.69%. The participants in the nonsurvival group had higher serum VA levels than those in the survival group (2.18 ± 0.82 vs. 2.78 ± 1.24 μmol/L, *p* < 0.01). Serum VA concentrations were positively correlated with serum creatinine levels (r = 0.56, *p* < 0.01) and urea nitrogen (r = 0.58, *p* < 0.01) but negatively correlated with eGFR (r = −0.56, *p* < 0.01). The serum VA level was independently related to all-cause mortality (hazard ratio (HR) = 1.15, [95% CI: 1.01–1.31], *p* = 0.03). The Kaplan–Meier survival analysis suggested that the survival probability was lower in participants with serum VA levels exceeding 2.09 μmol/L than in participants with serum VA levels below 2.09 μmol/L (*p* < 0.0001).

**Conclusion:**

A high serum VA was independently related to all-cause mortality in CKD patients. VA requirements for patients with CKD is worth studies in the future.

## 1 Introduction

Chronic kidney disease (CKD) is a progressive chronic condition characterized by a gradual decline in renal function (1). Approximately 10–15% of the world’s population suffers from CKD (2), affecting more than 25 million adults in the United States (3). The increasing incidence of CKD also represents a major public health burden in East Asia (4). In South Korea, the prevalence of CKD among individuals aged 20 and above is 8.2% (5). Annually, CKD is responsible for the deaths of 120 million people and results in 2.8 billion years of life lost (6). Furthermore, the global incidence of CKD is increasing (7–9). A study on the global kidney disease burden reported a 41.5% increase in CKD-related mortality from 1990 to 2017 (2). By 2040, CKD is projected to become the fifth leading cause of death worldwide, with the highest expected increase among all causes of death (10). In addition, CKD is also associated with comorbidities such as cardiovascular diseases and infections (11). Despite recent advancements in slowing disease progression and managing comorbidities, CKD patients are still at high risk of mortality (12). Early intervention and treatment are crucial for improving patient survival (13). Therefore, identifying biomarkers associated with CKD mortality is essential for early intervention in high-risk populations.

Vitamin A (VA), a group of fat-soluble micronutrients, including retinol, retinoic acid, and retinol ester, plays an important role in health (14, 15). Studies have reported that VA can serve as a nutritional indicator and is associated with the severity of the disease (16, 17). Recently, experimental studies have revealed that retinoic acid can alleviate kidney damage and inhibit kidney fibrosis (18–20). In addition, the kidneys play a key role in maintaining VA homeostasis in the body. Retinol is filtered by the renal glomerulus, with over 99% being reabsorbed by the proximal tubules (21). Animal studies have shown that impaired renal function can increase urinary retinol excretion (22). Whether the serum VA is related to long-term outcomes in the CKD population remains unclear. The aim of this study was to investigate the association between serum VA levels and long-term outcomes in patients with CKD. In this study, we found that higher serum VA correlated with lower eGFR, and higher serum creatinine and urea nitrogen in the CKD population. Furthermore, high serum VA levels were associated with increased all-cause mortality in the CKD population.

## 2 Materials and methods

### 2.1 Study population

The National Health and Nutrition Examination Survey (NHANES) is a population-based cross-sectional survey that investigates epidemiological information about the health and nutritional status of the US population. The survey collected data through interviews at home, physical examinations in mobile centers, and laboratory tests. The National Centre for Health Statistics (NCHS) ethics review board approved the NHANES protocol, and the data provided by the NHANES were anonymized and publicly available. All participants provided informed consent. Detailed information about the NHANES can be found at https://www.cdc.gov/nchs/nhanes.

**Figure 1:**
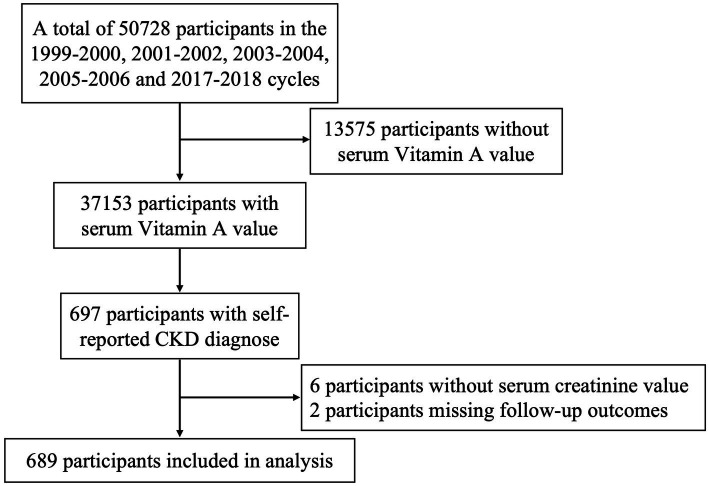
The flow chart of study participants.

In this study, we screened CKD participants who underwent serum VA level testing between 1999 and 2018. Serum VA levels were measured in the 1999–2000, 2001–2002, 2003–2004, 2005–2006, and 2017–2018 cycles, with a total of 37,153 participants. Based on self-reported CKD diagnoses, we identified 697 participants. Those without serum creatinine levels and those with missing follow-up data were excluded, resulting in a final sample of 689 participants. The data collection flow is illustrated in Figure 1.

### 2.2 Data collection

We collected demographic characteristics such as sex, age, and race, as well as socioeconomic factors such as education level (less than 9th grade, 9–11th grade, high school graduate/GED or equivalent, some college or AA degree, college graduate or above) and the ratio of family income to poverty (dividing Family Income by the Poverty Threshold). Self-reported dialysis history within the past 12 months was collected. Body mass index (BMI) was calculated by dividing the weight (in kg) by the squared height (in m^2^). All serum specimens were processed, stored, and shipped to the Division of Laboratory Sciences in National Center for Environmental Health for analysis. Laboratory indicators, including serum creatinine (Cr), blood urea nitrogen (BUN), and serum VA levels, were also collected. The estimated glomerular filtration rate (eGFR) was calculated using the four-variable modification of diet in renal disease (MDRD) equation (23). Additionally, we collected data on dietary VA intake. Dietary intakes of vitamin A were calculated using USDA’s Food and Nutrient Database for Dietary Studies 2017–2018 (FNDDS 2017–2018).

### 2.3 Primary outcome

The primary outcome was all-cause mortality among CKD patients. The all-cause mortality rate data for the NHANES cohort came from the NCHS records prior to December 31, 2019.

### 2.4 Statistical analysis

Normally distributed continuous data are represented by the mean ± standard deviation (SD), while nonnormally distributed data are represented by the interquartile range. Categorical variables are represented by frequencies and percentages. Student’s *t* test and the Kruskal–Wallis H test were used to compare continuous variables, while the chi–square test was used to compare categorical variables. We used 3 Cox regression models to evaluate the associations between serum VA levels and all-cause mortality among CKD patients. Model 1 was an unadjusted crude model. Model 2 was adjusted for age, sex, and race. In Model 3, we adjusted for age, sex, race, education level, and serum creatinine. The detailed associations between serum VA levels and risk of mortality were assessed by restricted cubic spline curves based on Cox regression models. The number of knots was set as 4 in the current study because 4 knots are a good compromise between flexibility and overfitting and could provide sufficient fit of the model (24).

We divided the CKD participants into two groups: the group with serum vitamin A levels less than 2.09 μmol/L (the upper limit of normal range of serum vitamin A) and the group with serum VA levels greater than 2.09 μmol/L. Kaplan–Meier (KM) curves were used to assess differences in survival between the two groups of participants with CKD. Pearson correlation was performed to analyze the correlation between serum VA levels and serum creatinine (Cr), blood urea nitrogen (BUN), and eGFR. We also analyzed the association between serum VA levels and all-cause mortality in CKD patients who received dialysis treatment.

All the statistical analyses were performed using R software (version 4.3.1). A two-tailed *p* value <0.05 was considered to indicate statistical significance.

## 3 Results

**Figure 2:**
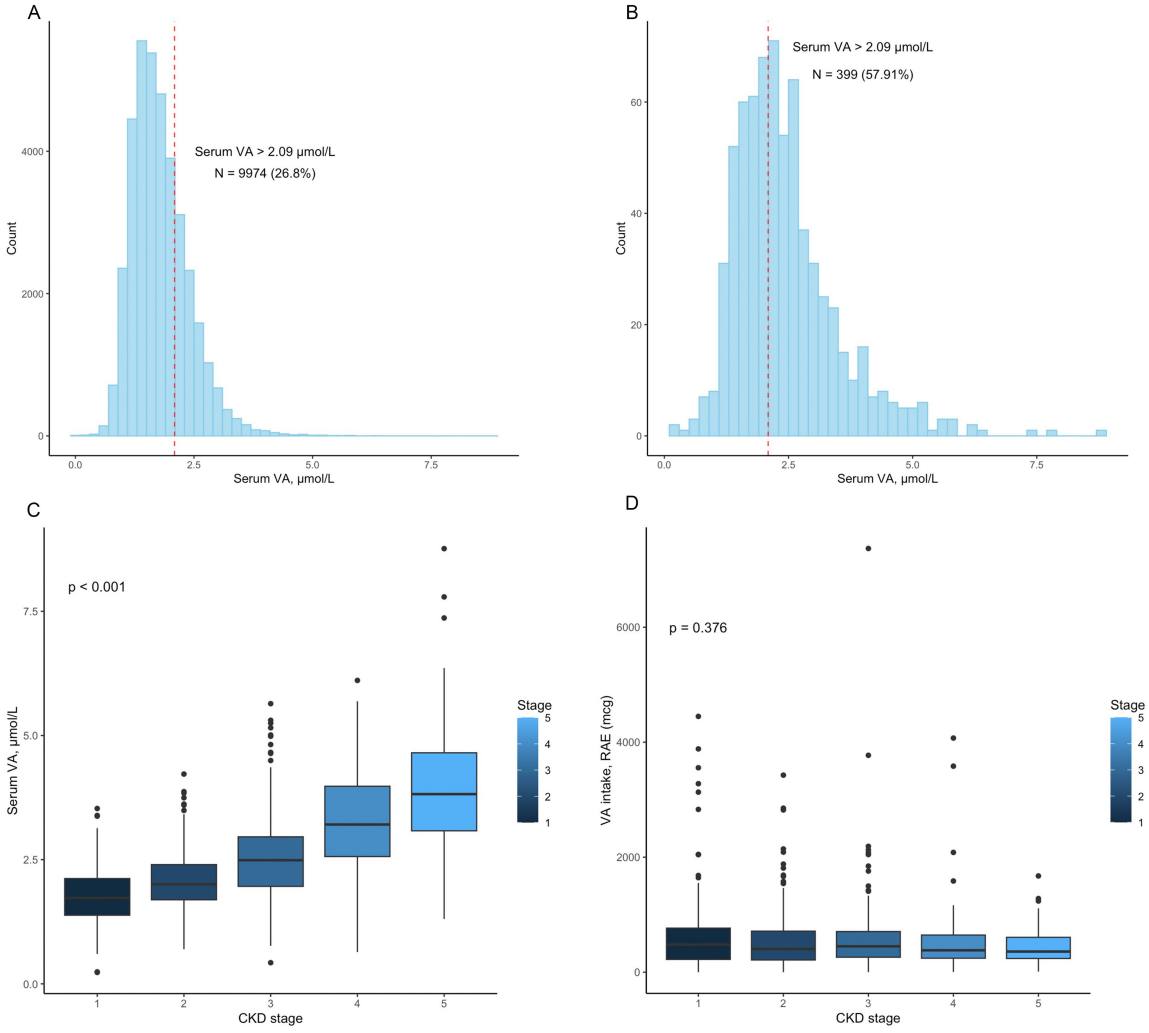
**(A)** The distribution of vitamin A levels in the 37153 participants. A total of 9974 (26.80%) patients had vitamin A levels that exceeded 2.09 μmol/L; **(B)** The distribution of vitamin A levels in the 689 participants. A total of 399 (57.91%) patients had vitamin A levels that exceeded 2.09 μmol/L; **(C)** Serum vitamin A levels in patients with different CKD stages (*p* < 0.001); **(D)** Vitamin A intake in patients with different CKD stages (*p* = 0.376).

The baseline characteristics are shown in Table 1. Among the 689 participants, the median age was 64.0 (50.0, 75.0) years. The serum VA level was 2.45 ± 1.06 μmol/L. Serum VA levels exceeded 2.09 μmol/L in 399 (57.91%) participants (Figure 2B). Serum VA concentrations gradually increased with progression of chronic kidney disease (Figure 2C). The dietary intake of VA was 436.0 (229.0, 710.0) mcg. By the end of the follow-up, a total of 301 participants had died, with a mortality of 43.69%. The age of the nonsurvival group was greater than that of the survival group (70.0 (61, 79) vs. 57 (43, 69.25) years, *p* < 0.01). There were significant differences between the two groups in terms of sex, race, and education level. There were no significant differences between the two groups in terms of family income to poverty, BMI, and VA intake. The eGFR in the nonsurvival group was significantly lower than that in the survival group (52.21 ± 35.18 vs. 74.85 ± 38.37 mL/min/1.73 m^2^, *p* < 0.01). The participants in the nonsurvival group had higher serum VA levels than those in the survival group (2.78 ± 1.24 vs. 2.18 ± 0.82 μmol/L, *p* < 0.01).

**Table 1:** Baseline characteristics of the 689 participants with CKD.

	All *N* = 689	Survival 388 (56.31%)	Non-survival 301 (43.69%)	*p*
Age, years	64.0 (50.0,75.0)	57.0 (43.0, 69.25)	70.0 (61.0, 79.0)	0.00^*^
Female, *n* (%)	348 (50.51%)	223 (57.47%)	125 (41.53%)	0.00
Race, *n* (%)				
Mexican American	130 (18.84%)	78 (20.10%)	52 (17.22%)	0.00
Other Hispanic	32 (4.64%)	27 (6.96%)	5 (1.66%)	
Non-Hispanic White	299 (43.33%)	148 (38.14%)	151 (50.0%)	
Non-Hispanic Black	186 (26.96%)	102 (26.29%)	84 (27.82%)	
Other Race	43 (6.23%)	33 (8.51%)	10 (3.31%)	
Ratio of family income to poverty	1.65 (0.99, 2.89)	1.69 (1.03, 3.25)	1.60 (0.96, 2.60)	0.33
Education, *n* (%)				
Less than 9th grade	148 (21.48%)	60 (15.464%)	88 (29.24%)	0.00
9-11th grade	124 (18.0%)	69 (17.784%)	55 (18.27%)	
High school graduate/GED or equivalent	147 (21.33%)	86 (22.165%)	61 (20.27%)	
Some college or AA degree	191 (27.72%)	121 (31.186%)	70 (23.26%)	
College graduate or above	79 (11.67%)	52 (13.402%)	27 (8.97%)	
History of dialysis, *n* (%)	56 (8.67%)	16 (4.29%)	40 (14.65%)	0.00
Serum creatinine, μmol/L	97.24 (70.72, 159.12)	83.54 (61.90, 125.75)	123.80 (79.60, 203.30)	0.00
Blood urea nitrogen, mmol/L	6.78 (4.60, 10.0)	5.71 (4.28, 8.21)	8.21 (5.70, 12.50)	0.00
eGFR, mL/min per 1.73 m^2^	64.96 ± 38.65	74.85 ± 38.37	52.21 ± 35.18	0.00
CKD stage, *n* (%)				
Stage 1	170 (24.67%)	126 (32.47%)	44 (14.62%)	0.00
Stage 2	176 (25.54%)	117 (30.15%)	59 (19.60%)	
Stage 3	217 (31.49%)	105 (27.06%)	112 (37.21%)	
Stage 4	67 (9.72%)	25 (6.44%)	42 (13.95%)	
Stage 5	59 (8.56%)	15 (3.87%)	44 (14.62%)	
BMI, kg/m^2^	30.51 ± 7.87	30.97 ± 8.08	29.88 ± 7.55	0.08
Dietary intake of VA, mcg	436.0 (234.2, 710.2)	444.0 (222.0, 720.0)	420.50 (246.25, 697.37)	0.94
Serum VA, μmol/L	2.45 ± 1.06	2.18 ± 0.82	2.78 ± 1.24	0.00

CKD, chronic kidney disease; VA, Vitamin A; ^*^p < 0.01.

**Figure 3:**
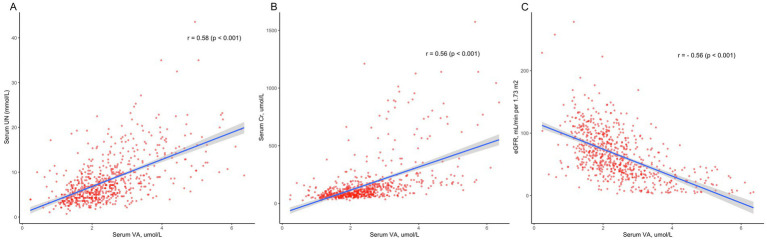
The correlation between serum vitamin A levels and kidney function. **(A)** Serum vitamin A levels were positively correlated with serum creatinine (Cr) levels (r = 0.56, *p* < 0.01). **(B)** Serum vitamin A levels were positively correlated with urea nitrogen (UN; r = 0.58, *p* < 0.01). **(C)** Serum vitamin A concentrations were negatively correlated with the eGFR (r = −0.56, *p* < 0.01).

We performed Pearson correlation to explore the correlation between serum VA levels and creatinine, urea nitrogen, and eGFR (Figure 3). The results showed that serum VA concentrations were positively correlated with serum creatinine levels, with a correlation coefficient of 0.56 (*p* < 0.01). VA levels were also positively correlated with urea nitrogen, with a correlation coefficient of r = 0.58 (*p* < 0.01). However, the serum vitamin A concentration was negatively correlated with the eGFR, with a correlation coefficient of r = −0.56 (*p* < 0.01).

**Figure 4:**
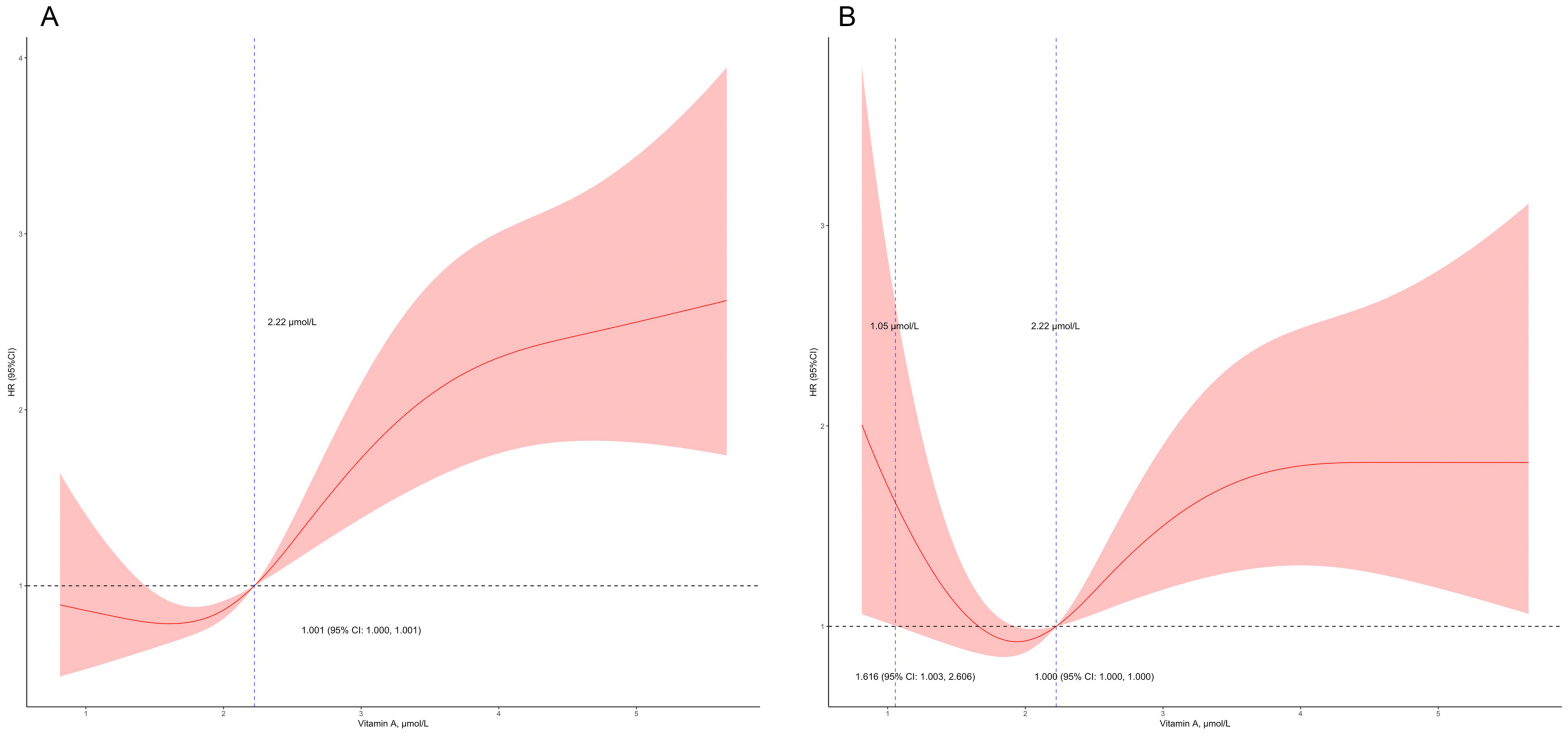
Detailed association between serum vitamin A levels and mortality. **(A)** Vitamin A levels >2.22 μmol/L was related to increased risk of mortality; **(B)** Vitamin A levels >2.22 μmol/L and <1.05 μmol/L were related to increased risk of mortality in the adjusted model (adjusted for age, sex, race, education level, and serum creatinine).

We conducted univariate Cox regression analysis (Table 2), and the results showed that serum VA levels were related to mortality (hazard ratio (HR) = 1.37, 95% confidence interval (CI): 1.26–1.50, *p* < 0.01). Sex, age, race, education level and serum creatinine were related to all-cause mortality in the univariate analysis. After adjustment for age, sex, and race in the model 2, serum VA levels were associated with increased risk of mortality [HR = 1.24 (95% CI: 1.13–1.37)]. In the model 3, the serum VA level was independently related to all-cause mortality, with a HR = 1.15 (95% CI: 1.01–1.31, *p* = 0.03). In the restricted cubic spline analysis, VA levels >2.22 μmol/L were associated with increased risk of mortality in the rude model (Figure 4A). In the adjusted cubic spline, VA levels >2.22 μmol/L and <1.05 μmol/L were associated with increased risk of mortality (Figure 4B).

**Table 2:** Cox regression analyses for association between factors and all-cause mortality.

	Univariate analysis		Multivariate analysis	
	HR (95% CI)	*p*	HR (95% CI)	*p*
Gender^a^	0.53 (0.42–0.67)	0.00^*^	0.73 (0.57–0.95)	0.02
Age	1.06 (1.05–1.07)	0.00	1.06 (1.05–1.07)	0.00
Race^b^	1.57 (1.25–1.97)	0.00	1.18 (0.89–1.57)	0.24
BMI	0.99 (0.97–1.01)	0.08	1.00 (0.98–1.02)	0.95
Education^c^	0.91 (0.83–0.98)	0.02	0.95 (0.86–1.04)	0.23
Serum creatinine	1.13 (1.09–1.16)	0.00	1.09 (1.04–1.15)	0.00
Serum VA	1.37 (1.26–1.50)	0.00	1.15 (1.01–1.31)	0.03

HR, hazard ratio; CI, confidence interval; VA, vitamin A. ^*^p < 0.01.

a Male is the reference.

b Non-Hispanic White was the reference.

c Less than 9th grade was the reference.

**Figure 5:**
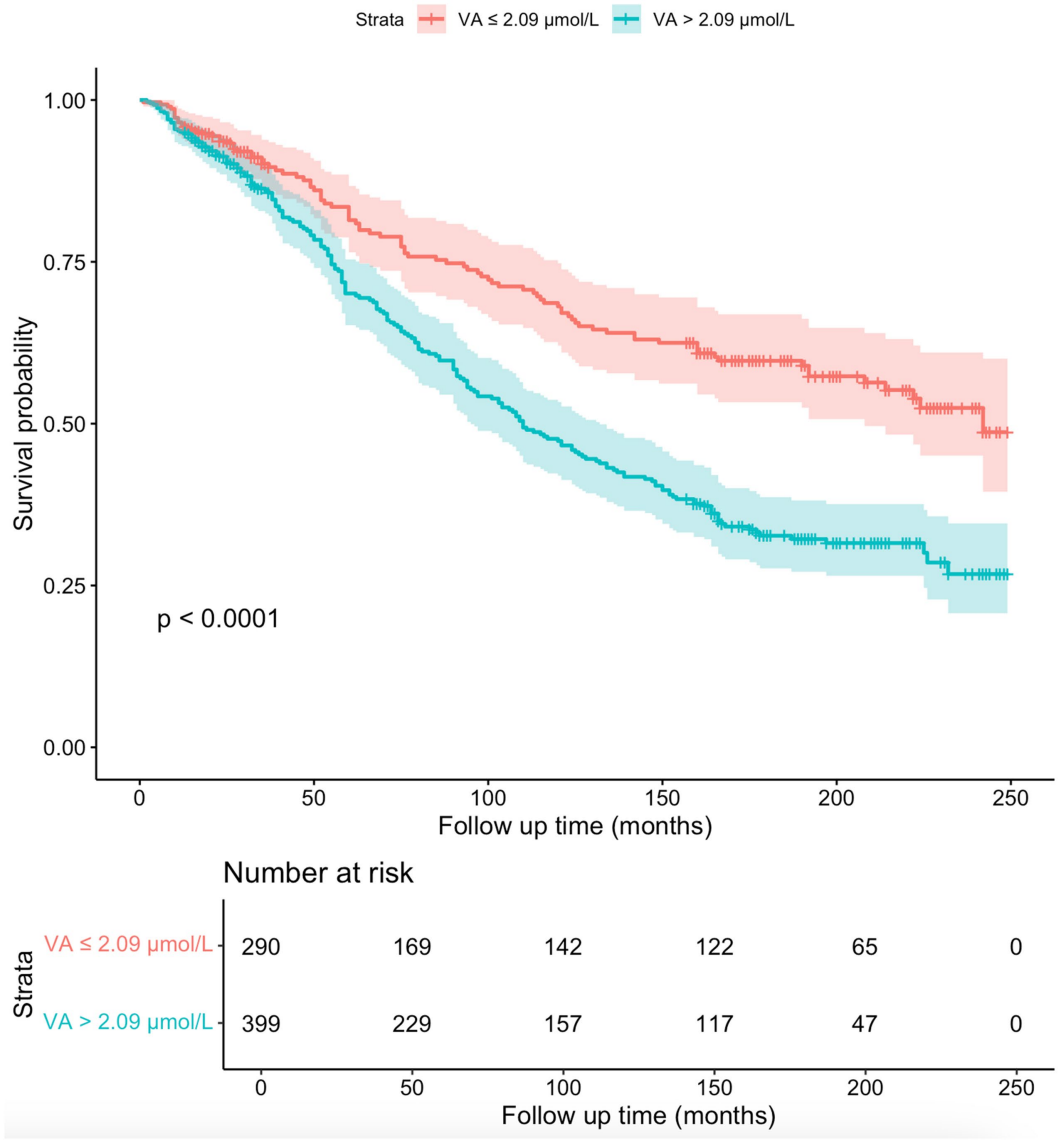
The Kaplan–Meier survival curves for the 689 participants. The survival probability was lower in participants with serum VA levels exceeding 2.09 μmol/L than in participants with serum VA levels below 2.09 μmol/L (*p* < 0.0001).

Figure 5 shows the Kaplan–Meier survival curves for participants with serum VA levels below 2.09 μmol/L and participants with serum VA levels exceeding 2.09 μmol/L. The results of the KM analysis suggested that the survival probability was lower in participants with serum VA levels exceeding 2.09 μmol/L than in participants with serum VA levels below 2.09 μmol/L. According to the log-rank test, this difference was highly statistically significant (*p* < 0.0001).

**Figure 6:**
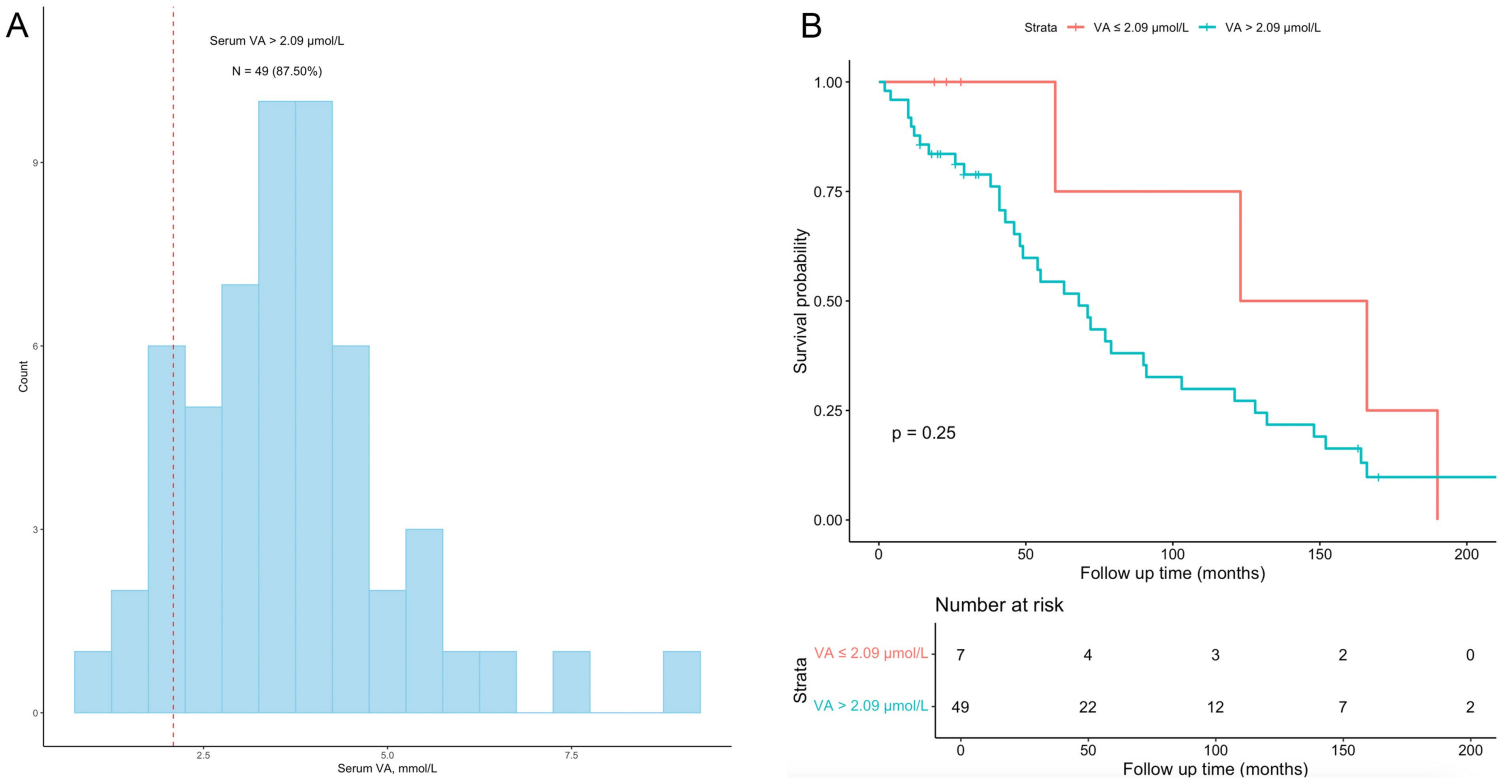
Subgroup analyses among CKD patients with a history of dialysis. **(A)** the distribution of vitamin A levels among the 56 participants with a history of dialysis; 49 (87.50%) had vitamin A levels exceeding 2.09 μmol/L. **(B)** Kaplan–Meier survival curves for the 56 participants; the difference in survival probability was not significant between participants with serum vitamin A levels exceeding 2.09 μmol/L and participants with serum vitamin A levels below 2.09 μmol/L (*p* = 0.25).

We performed a subgroup analysis of CKD patients who received dialysis treatment. Fifty-six (8.67%) participants had a history of dialysis within the past 12 months (Table 1). The characteristics of the 56 patients are shown in Table 3. All-cause mortality was 71.43%. A total of 87.50% of participants had a serum VA level exceeding 2.09 μmol/L (Figure 6). The serum VA level in participants with a history of dialysis (3.66 ± 1.45 μmol/L) was much greater than that in the CKD population (2.45 ± 1.06 μmol/L). The serum VA level in the survival group was slightly greater than that in the nonsurvival group (3.78 ± 1.46 vs. 3.61 ± 1.46 μmol/L), but the difference was not significant. Among the 56 participants, the difference in survival probability was not significant between participants with serum vitamin A levels exceeding 2.09 μmol/L and participants with serum vitamin A levels below 2.09 μmol/L (*p* = 0.25).

**Table 3:** The characteristics of the 56 CKD participants with History of dialysis.

	All *N* = 56	Survival 16 (28.57%)	Non-survival 40 (71.43%)	*p*
Age, years	61.0 (50.0, 68.25)	63.50 (51.50, 67.50)	60.50 (50.0, 68.25)	0.55
Female, *n* (%)	18 (50.51%)	4 (25.0%)	14 (35.0%)	0.68
Race, *n* (%)				
Mexican American	11 (19.64%)	2 (12.50%)	9 (22.50%)	0.02
Other Hispanic	2 (3.57%)	1 (6.25%)	1 (2.50%)	
Non-Hispanic White	12 (21.43%)	5 (31.25%)	7 (17.50%)	
Non-Hispanic Black	28 (50.0%)	5 (31.25%)	23 (57.50%)	
Other Race	3 (5.36%)	3 (18.75%)	0 (0.0%)	
Education, *n* (%)				
Less than 9th grade	11 (19.64%)	3 (18.75%)	8 (20.0%)	0.17
9-11th grade	12 (21.43%)	2 (12.50%)	10 (25.0%)	
High school graduate/GED or equivalent	11 (19.64%)	1 (6.25%)	10 (25.0%)	
Some college or AA degree	15 (26.79%)	8 (50.0%)	7 (17.50%)	
College graduate or above	7 (12.50%)	2 (12.50%)	5 (12.50%)	
Vitamin A intake, mcg	335.0 (195.25, 625.0)	338.0 (169.0, 753.0)	405.0 (222.0, 600.50)	0.97
Serum Vitamin A, μmol/L	3.66 ± 1.45	3.78 ± 1.46	3.61 ± 1.46	0.71

Student’s *t* test, Kruskal–Wallis H test, and chi–square test were used for comparison between groups.

## 4 Discussion

This study analyzed the association between serum VA levels and all-cause mortality in CKD patients in the NHANES database. The results showed that the serum VA level in the nonsurvival group of CKD patients was greater than that in the survival group. Correlation analysis revealed that the serum VA was negatively correlated with the eGFR and positively correlated with the creatinine and urea nitrogen levels. Serum VA levels were independently related to all-cause mortality with nonlinear pattern among the CKD population.

The kidney plays an important role in maintaining the homeostasis of serum VA levels. Retinol can be filtered out of the glomerulus, after which most of the retinol is reabsorbed by the proximal renal tubules. Animal experiments have revealed that damage to the proximal renal tubules can increase the concentration of retinol binding protein and retinol in the urine, leading to increase in loss of retinol (25). However, the serum VA level in our study population was 2.45 ± 1.06 μmol/L, higher than the recommended upper limit of the serum VA level (2.09 μmol/L) (14). More than half of the participants had serum VA levels greater than 2.09 μmol/L, while there is only 12% of NHANES general population had serum VA levels exceeding 2.09 μmol/L (26), suggesting that renal function insufficiency can lead to an increase in serum VA levels. CKD patients often suffer from glomerular damage (27). Although tubular damage can lead to excessive loss of VA, glomerular damage prevents serum VA from being filtered through the glomerulus, leading to the accumulation of VA in serum (28). In our study, we found that the serum VA level was negatively correlated with the eGFR (r = −0.56) and positively correlated with the creatinine and urea nitrogen levels, suggesting that the increase in the circulating VA level is closely related to the decline in glomerular filtration function.

Previous studies have explored the relationship between serum VA levels and the outcome of CKD patients (29). The study followed up 261 dialysis CKD patients for 5 years, and showed that the serum VA level was increased in CKD patients, which is consistent with the results of our study. However, the study revealed that a low serum vitamin A level was an independent predictor of poor prognosis, and a high vitamin A level was related to a lower all-cause mortality risk (HR = 0.733, 95% CI: 0.599–0.896) and cardiovascular mortality risk (HR = 0.694, 95% CI: 0.511–0.942), which is different from our study. In our study, we found that higher serum VA was independently related to all-cause mortality in the CKD population. In addition, we identified participants who received dialysis treatment and found that the serum VA level in the survival group was slightly lower than that in the nonsurvival group (3.78 ± 1.46 vs. 3.62 ± 1.47 μmol/L). Though survival analysis did not reveal a relationship between the serum vitamin A concentration and mortality in participants who received dialysis, the significance of high serum VA levels in dialysis CKD patients may be different from that in nondialysis CKD patients.

In this study, the serum VA level in the nonsurvival group was greater than that in the survival group in the CKD population, but there was no significant difference in the amount of VA intake between the two groups, suggesting that the possible explanation for the increase in the circulating VA level in CKD patients was VA accumulation. Although experimental studies have revealed that retinoic acid can alleviate kidney damage and inhibit kidney fibrosis (18–20), higher VA levels are not associated with better outcomes in this study. More importantly, the serum VA levels in most CKD patients were greater than the upper limit of the recommended level of 2.09 μmol/L, suggesting that the amount of VA in the circulation among CKD patients may exceed tissue needs. Currently, optimal serum VA levels, as well as amounts of daily VA intake, have not been defined for CKD patients (30). As hypervitaminosis A has potential toxicity (31), vitamin A requirements for patients with CKD is worth studies in the future.

This study has several limitations. First, the diagnosis of CKD was based on patient self-reports, which may not be accurate. Second, the date of CKD onset cannot be determined in the NHANES database. Whether a long duration of CKD leads to a high serum VA level should be further studied. Third, we revealed that high serum levels may differentially affect the outcomes of dialysis CKD patients and nondialysis CKD patients. However, there are few cases of dialysis. We failed to reveal a significant relationship between serum vitamin A levels and outcomes in CKD patients with a history of dialysis.

## 5 Conclusion

In conclusion, this study revealed that serum VA levels in CKD patients were correlated with kidney function. Higher serum VA levels were correlated with lower eGFRs and higher serum creatinine and urea nitrogen levels in the CKD population. Furthermore, a high serum VA was independently related to all-cause mortality in CKD patients. Whether the recommended daily intake of VA needs to be adapted for CKD patients requires further study.

## Data Availability

The datasets presented in this study can be found in online repositories. The names of the repository/repositories and accession number(s) can be found below: https://www.cdc.gov/nchs/nhanes.
